# Mechanical Response Analysis of Ultra-Thin Asphalt Wearing Course Pavement Under Non-Uniform Loading Pressure

**DOI:** 10.3390/ma18143335

**Published:** 2025-07-16

**Authors:** Wei Zhou, Yingying Dou, Chupeng Chen, Yi Yang, Xinquan Xu, Lintao Li, Jiangyin Xiao, Feng Chen

**Affiliations:** 1 Guangdong Provincial Transportation Industrial Investment Co., Ltd., Guangzhou 510623, China; zhouhj1109@163.com; 2 Guangdong Hualu Transport Technology Co., Ltd., Guangzhou 510420, China; yitoudataiyang@outlook.com (Y.D.); xjy_ow@163.copm (C.C.); xuxinquan998@126.com (X.X.);; 3 School of Transportation, Southeast University, Nanjing 211189, China; fengc@seu.edu.cn

**Keywords:** asphalt pavement, ultra-thin wearing course, finite element analysis, non-uniform load, influence factor

## Abstract

Traditional ultra-thin asphalt wearing course designs often oversimplify wheel loads as uniform pressures, neglecting critical non-uniform effects. This study establishes a 3D finite element model incorporating realistic non-uniform tire loading to reveal its mechanistic influence on pavement responses. Results demonstrate that non-uniform loading significantly alters stress states in ultra-thin layers, substantially elevating critical stresses compared to uniform assumptions. A novel Non-uniform Load Influence Factor (NLIF) accounting for thickness effects is developed to quantify these deviations. The analysis provides a foundation for revising material strength specifications and fatigue design criteria, contributing to improved performance and durability of ultra-thin pavement systems.

## 1. Introduction

As a novel preventive maintenance technology, ultra-thin wearing courses are suitable for maintenance of asphalt pavements with heavy traffics and high-performance requirements, and can also be applied as surface wearing course for newly constructed pavements [1,2]. Compared to traditional wearing course layer with a thickness of 4–5 cm, ultra-thin wearing courses typically range from 1.5–2.5 cm in thickness, featuring convenient construction, economical material usage, and excellent functional characteristics [3]. Research has shown that properly designed ultra-thin wearing courses not only provide good skid resistance and surface texture depth, but also effectively reduce traffic noise, significantly improving pavement performance [4,5].

Currently, ultra-thin wearing course design adopts mostly the standard axle load dual-wheel uniform load assumption. However, the actual contact stress distribution between tires and pavement is much more complex than that induced by uniform loading [6]. Studies have shown that tire contact pressure distribution is influenced by multiple factors, including vertical axial load, tire pressure, tire tread pattern, and pavement surface texture. Through finite element modelling and analyses, Zheng [7] found that under different non-uniform loads, surface deflection and tensile stress at the bottom of asphalt layers showed a linear growth trend, with stress levels under overload conditions higher than those under design axle loads. Ye [8] pointed out that under non-uniform loading, the maximum shear stress occurs in the gaps between tire tread bars and moves toward the tire center as axle load increases, exhibiting stress distribution characteristics significantly different from uniform load assumptions. With the reduced thickness of ultra-thin wearing courses, these non-uniform load effects may become more significant, causing local material stresses far exceeding design expectations and accelerating structural performance degradation. Therefore, it is necessary to conduct in-depth research on stress distribution patterns and influencing factors of ultra-thin wearing courses under non-uniform loading conditions.

Early works on ultra-thin wearing course material were found in France. In that, Bellanger [9] systematically summarized the technical progress in reducing asphalt layer thickness in France during the 1980s–1990s, proposing the concept of gap-graded design. The technology subsequently experienced rapid international development. In Europe, Sandberg et al. [10] conducted a comprehensive transnational ERA-NET ROAD project on “Optimization of thin asphalt layers”, providing systematic evaluation of thin asphalt mixtures up to 30 mm thickness and establishing fundamental design principles. Meanwhile, European researchers advanced mechanical response modeling approaches, with De Visscher et al. [11] developed sophisticated testing protocols including ARTe and T2R methods to simulate traffic-induced shear forces on ultra-thin wearing courses. In the United States, Mogawer et al. [12] investigated the effect of binder modification on ultra-thin overlay performance, demonstrating significant improvements in pavement preservation strategies. However, existing research has mainly focused on material optimization rather than systematic investigation of mechanical response characteristics under non-uniform loading conditions, particularly the complex stress distributions that may significantly impact ultra-thin layer performance [13].

Ultra-thin wearing course technology started late in China, but has developed rapidly and different mixture types have emerged. Liu [14] demonstrated through systematic testing and evaluation that ultra-thin asphalt overlays can significantly improve pavement smoothness, skid resistance, and frost resistance when using reasonable material design and construction techniques. Zhang et al. [15] investigated the application effects of epoxy-modified asphalt and steel slag ultra-thin friction courses in highway maintenance and proposed optimized curing protocols and material formulations to enhance skid resistance, durability, and pavement performance. Cui [16] optimized the gradation design of ultra-thin wearing course asphalt using the Course Aggregate Void Filling method, demonstrating superior high-temperature rutting resistance, skid durability, and comprehensive performance in low-temperature crack and water stability compared to traditional mixtures. Yu [3] conducted a comprehensive review of high-performance ultra-thin wearing course technology, systematically summarizing key technical points in material selection, mix design, and construction techniques. However, existing research has mainly focused on improvements on material mix optimization and construction technique, with limited research on the mechanical response characteristics and influence mechanisms of ultra-thin wearing courses under traffic loading, especially the non-uniformity of load pattern which could have most detrimental effect on the wearing course layer. In particular, the non-uniform load effect with respect to the changes in thickness of wearing course layer, as well as the differences in sensitivity to non-uniform loads among different types of mixture gradation, still lack systematic theoretical understandings.

This paper aims to employ a finite element modelling approach to gain a deeper insight into the ultra-thin wearing course pavement structure considering the vital influence from non-uniformity of tire loading pressure. By comparing the pavement internal stress distribution characteristics under uniform and non-uniform loading conditions, the influence patterns of non-uniform loads on the mechanical response of ultra-thin wearing course layer and underlying structural layers are revealed. A Non-uniform Load Influence Factor (NLIF) is further established, and based on which, corrections are proposed for material strength test and fatigue prediction model of ultra-thin wearing course. The research findings provide theoretical guidance for the structural design and optimization of ultra-thin wearing course pavement.

## 2. Finite Element Modeling

### 2.1. Model Construction

**Figure 1:**
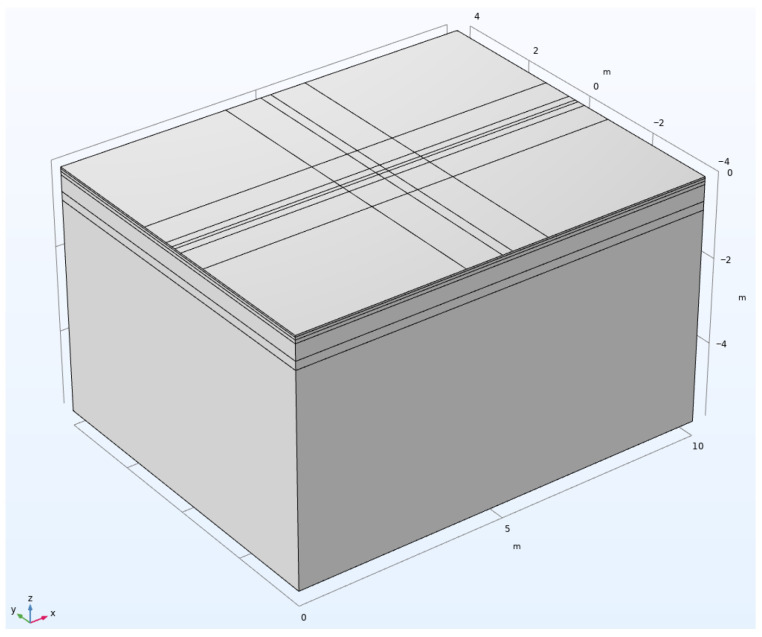
Geometric model.

**Figure 2:**
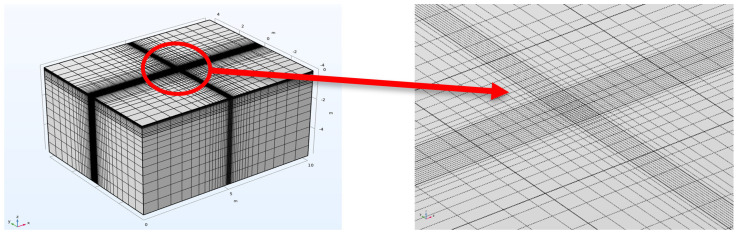
Mesh refinement.

A three-dimensional asphalt pavement finite element numerical simulation model was established using COMSOL finite element software version 6.2, with the model geometry shown in Figure 1, which is a typical semi-rigid base asphalt pavement structure. This model was established based on the plane stress assumption, with parameter values of thickness, material modulus, and Poisson’s ratio for each structural layer shown in Table 1. Notably, the elastic modulus were derived from field measurements compliant with *Specifications for Design of Highway Asphalt Pavement (JTG D50-2017)* [17], while Poisson’s ratios adhere to Section 5.6 (Poisson’s Ratio) of the same specification—including the 0.25 value prescribed for both inorganic stabilized materials and dense-graded asphalt mixtures. Geometric boundary conditions were set as follows: continuity between layers, fixation at the bottom and lateral sides. The mesh as shown in Figure 2, was refined around the loading areas.

The finite element model is based on the fundamental principles of solid mechanics. The analysis solves for the stress and strain fields that satisfy mechanical equilibrium throughout the pavement structure under the applied loads.

A key aspect of the model is the use of appropriate constitutive laws (material models) to describe the behavior of each layer:

(1) For the asphalt layers (Ultra-thin, Surface, Middle, and Bottom layers), a viscoelastic material model was used. This model accurately captures the time-dependent nature of asphalt concrete, where the stress at any given moment is a function of the entire history of strain. This is essential for modeling phenomena such as stress relaxation and creep.
(2) For the cement-stabilized base layers (Upper Base, Lower Base), a linear elastic material model was employed. This model assumes a direct, time-independent linear relationship between stress and strain (Hooke’s Law), which is a standard and effective representation for these stiffer, unbound, or stabilized materials.

The COMSOL software numerically solves the governing equations based on these assumptions to compute the stress distribution within the multi-layered pavement system.

### 2.2. Load Boundary Conditions

**Figure 3:**
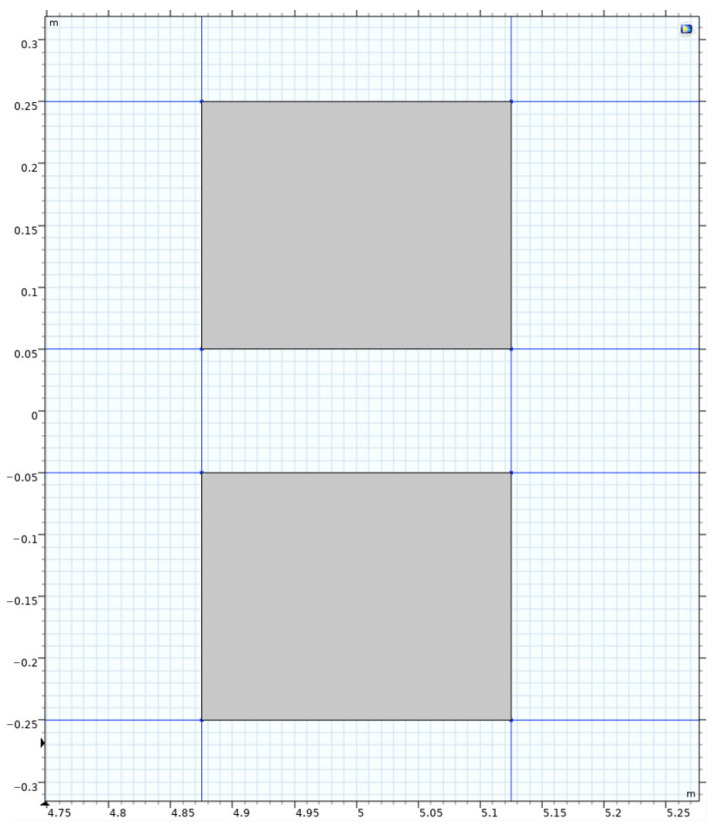
Load diagram.

**Figure 4:**
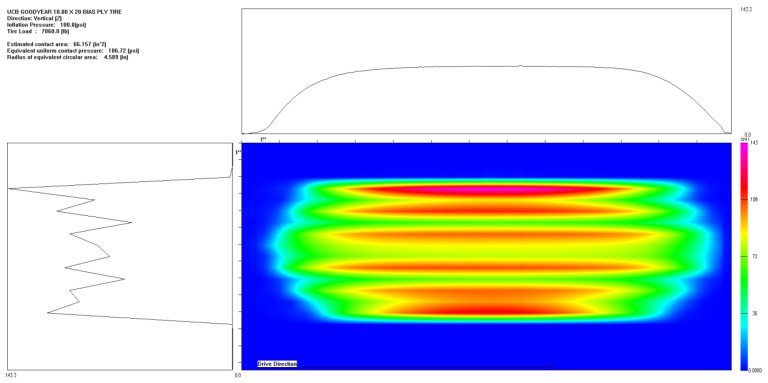
Tire load distribution diagram.

**Figure 5:**
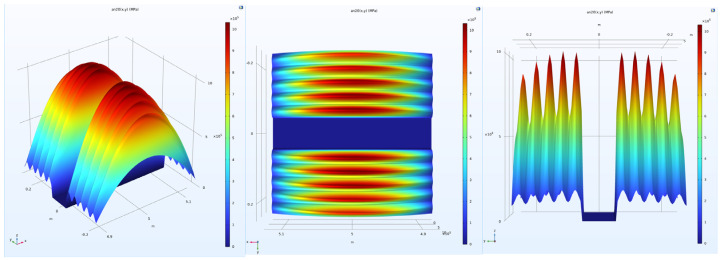
Non-uniform load simulation based on actual tire load distribution.

To investigate the effects of uniform and non-uniform loads on the mechanical simulation of ultra-thin wearing course asphalt pavement structures, both uniform and non-uniform loads were applied to the pavement structure, with simulations conducted under both constant speed and braking conditions. The specific load conditions were as follows:

(1) Uniform load: A standard dual-wheel axle load of 100 kN was adopted, determining the corresponding pressure as 0.707 MPa. According to the equivalence principle, the wheel load was simplified as a rectangular uniform load, with single wheel dimensions set as 0.2 m × 0.25 m and a center distance of 0.3 m between the two wheels, as shown in the dark area in Figure 3.
(2) Non-uniform load: To simulate the actual pressure distribution of tires on the pavement, the tire-pavement contact stress distributions were obtained from the TireView database developed by the Texas Transportation Institute. TireView provides comprehensive contact pressure distributions in longitudinal, lateral, and vertical directions for various tire types under different loading conditions [18]. The actual pressure distribution is shown in Figure 4. The non-uniform load simulated based on the actual pressure distribution was also applied within the range of 0.2 m × 0.25 m, with an equivalent average contact pressure of 0.736 MPa, and pressure peaks located at y = ±0.1085 m. The simulated load is shown in Figure 5.

During load application, there was a vertically downward axle weight and a horizontally backward rolling friction force. The rolling friction force was considered as the product of the axial force and the friction coefficient, with a friction coefficient of 0.05 at constant speed. During braking load application, there was a vertically downward axle weight and a horizontally forward horizontal braking force. The horizontal braking force was considered as the product of the axial force and the friction coefficient, with a friction coefficient of 0.5 during braking.

## 3. Comparative Analysis of Ultra-Thin Layer Pavement Structure Stress Under Uniform and Non-Uniform Loads

### 3.1. Vertical Stress

**Figure 6:**
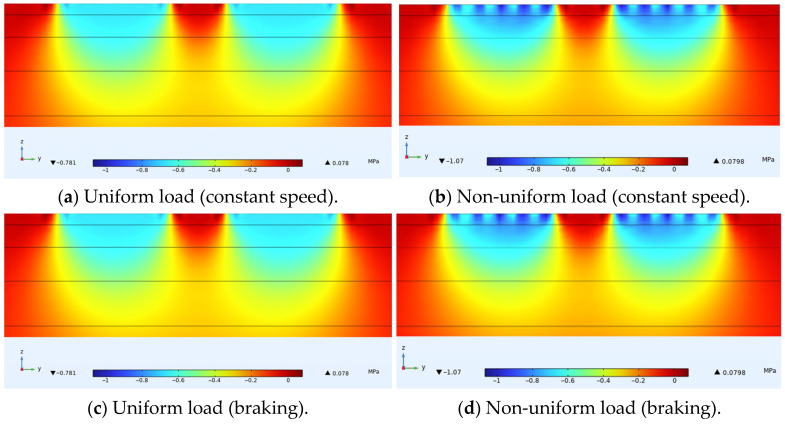
Internal vertical stress distribution in pavement structure.

Figure 6 shows the cross-sectional stress distribution passing through the tire center, comparing the internal vertical stress distribution of ultra-thin wearing course asphalt pavement structures under uniform and non-uniform loads. From the stress contour in Figure 6, it was observed that: (1) With similar equivalent average contact pressures for both load types, the extreme value of vertical stress on this cross-section under uniform load was only about 0.79 MPa (Figure 6a), while under non-uniform load, the extreme value of internal vertical stress in the pavement structure was about 1.07 MPa (Figure 6b), an increase of 35.4%, which indicated a significant difference. This was because tire pressure was directly averaged over the surface, neglecting the existence of tire pressure extreme values. (2) The influence of non-uniform loads was mainly concentrated in the asphalt thin layer, and it was found that simplifying the load as a rectangular uniform load could not reflect the actual load variation on the surface. (3) A comparison of Figure 6a with Figure 6c and Figure 6b with Figure 6d revealed that the magnitude of vertical stress was not affected by the horizontal braking force. This was likely because in the process of simplifying tire loads as uniform loads, the vertical stresses on this cross-section under constant speed and braking conditions for the same load form were almost identical.

Asphalt layer rutting is significantly affected by vertical stress. Using non-uniform loads for simulation can more specifically and intuitively analyze the location and development of rutting in the wearing layer and surface layer, as well as analyze the distribution of stress transfer to the underlying asphalt load-bearing layer. Such a significant increase in vertical compressive stress raised concerns about the pavement’s susceptibility to permanent deformation (rutting), which significantly shortens pavement life and triggers costly rehabilitation efforts.

### 3.2. Horizontal Transverse Stress

**Figure 7:**
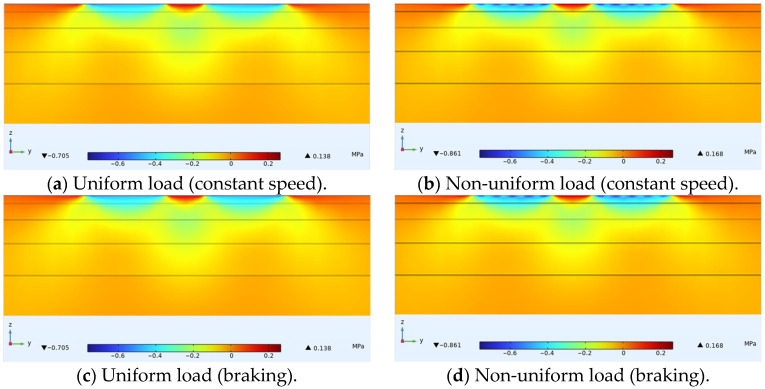
Internal horizontal transverse stress distribution in pavement structure.

Figure 7 shows the distribution of horizontal transverse stress within ultra-thin wearing course asphalt pavement structures under uniform and non-uniform loads, with a cross-sectional stress distribution passing through the tire center drawn for convenient display. Similar to the previous section, under the same load form, horizontal transverse stresses on this cross-section under constant speed and braking conditions were almost identical. Comparing Figure 7a with Figure 7b and Figure 7c with Figure 7d revealed that under non-uniform load, the extreme value of horizontal compressive stress on this cross-section was about 0.861 MPa, while under uniform load, the extreme value of horizontal compressive stress was about 0.705 MPa. This difference was also most evident within the ultra-thin wearing layer. The extreme values of horizontal tensile stress under load were located at the center of the wheel track and on both sides of the tire. Under non-uniform load, the extreme value was about 0.168 MPa, while under uniform load, the extreme value was about 0.138 MPa. Analysis of longitudinal cracks in the wearing layer caused by horizontal tensile stress indicated that the uniformity of the load had negligible impact on the results.

### 3.3. Horizontal Longitudinal Stress

**Figure 8:**
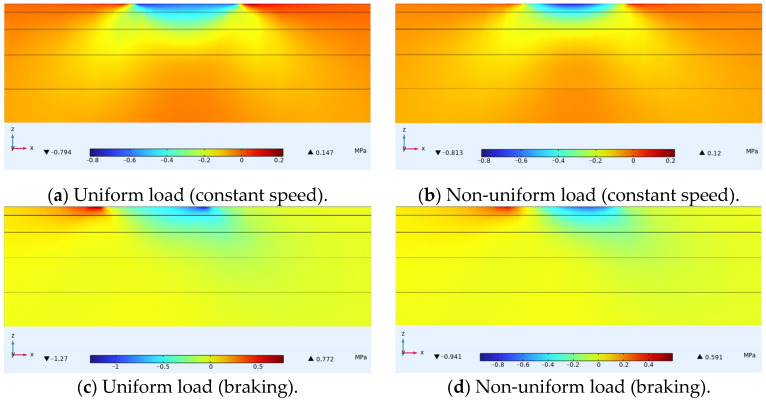
Internal horizontal longitudinal stress distribution in asphalt pavement structure.

Horizontal longitudinal stress is mainly related to transverse cracks in the structure. The study drew longitudinal stress distribution diagrams of cross-sections in the driving direction passing through the tire center under constant speed conditions, and cross-sections in the driving direction passing through the load extreme points under non-constant speed conditions, with the positive x-axis direction being the driving direction, as shown in Figure 8. It was found that under constant speed or braking conditions, there was no significant difference in the form of stress distribution produced within the cross-section between uniform and non-uniform loads. Focusing on the ultra-thin wearing layer, it was found that under constant speed non-uniform load, the extreme value of compressive stress produced within the tire load area was higher than that under constant speed uniform load, while the extreme value of tensile stress produced on both sides of the tire is lower. Under non-uniform load, the extreme value of compressive stress was 0.813 MPa and the extreme value of tensile stress was 0.120 MPa, as shown in Figure 8b. Under uniform load, the extreme value of compressive stress was 0.794 MPa and the extreme value of tensile stress was 0.147 MPa, as shown in Figure 8a. Under braking conditions, compressive stresses generated by non-uniform loads—both behind and ahead of the tire contact area—and the tensile stress behind the tire were all lower than those induced by uniform loads in the corresponding regions, as shown in Figure 8c,d. Under non-uniform loading, compressive stress within the wearing layer at the tire contact center exceeded that under uniform loading. This occurred because the actual contact pressure distribution follows a parabolic pattern, whereas uniform loading redistributes force equally across the contact area. Consequently, uniform loading overestimates edge stresses while underestimating central stresses due to averaging effects.

### 3.4. Shear Stress

**Figure 9:**
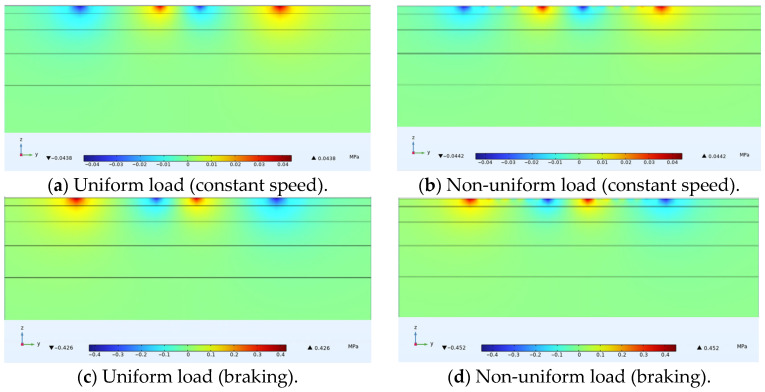
Internal shear stress distribution in pavement structure.

Figure 9 shows the distribution of shear stress within ultra-thin wearing course asphalt pavement structures under uniform and non-uniform loads, with a cross-sectional stress distribution passing through the tire center drawn. Under all four conditions, the stress distribution forms were basically the same, with several peaks of non-uniform loads causing relatively small shear stresses in the corresponding contact areas. Under constant speed or braking conditions, the magnitude of shear stress caused by uniform and non-uniform loads on both sides of the tire was basically the same.

## 4. Calculation and Analysis of Pavement Structure Stress Under Non-Uniform Constant Speed Loads with Different Thin Layer

Since the mechanical properties of ultra-thin wearing course materials differ somewhat from traditional asphalt mixtures, analyzing the effect of different thin layer thicknesses and moduli on asphalt pavement structure stress through pavement structure stress calculations can provide a mechanical basis for ultra-thin wearing course structural and material design.

### 4.1. Vertical Stress Under Varying Thin Layer Thicknesses

**Figure 10:**
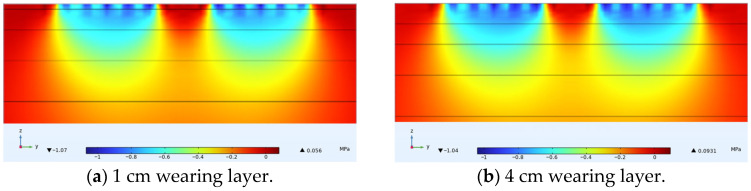
Internal vertical stress distribution in thin layer paved asphalt pavement structure under different thin layer thicknesses.

**Figure 11:**
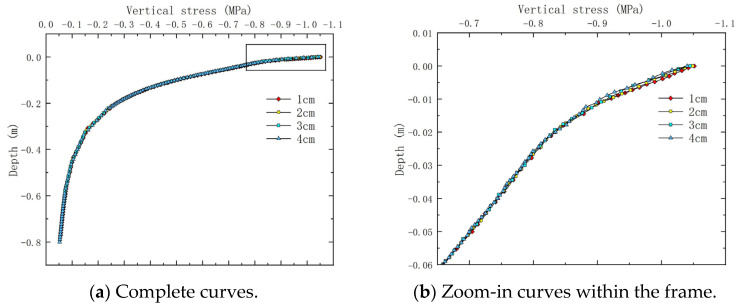
Vertical stress variation along pavement depth for different thin layer thicknesses (the rectangular frame in (**a**) indicates the zoomed region displayed in (**b**)).

Vertical stress is generally related to compaction rutting. Figure 10 shows the internal vertical stress distribution of ultra-thin wearing course asphalt pavement cross-sections under constant speed loading. Figure 11 shows the variation of vertical stress along the depth direction at load peak points when the thin layer thickness is 1, 2, 3, and 4 cm. For thin layer asphalt pavement structures, asphalt ultra-thin wearing layers can protect the underlying asphalt load-bearing pavement structure, reducing to some extent the permanent deformation of the asphalt pavement surface caused by traffic loads. As illustrated in Figure 10, the thickness of the wearing course plays a critical role in how these amplified stresses are transmitted to the underlying layers. Specifically, for thinner wearing courses, the high vertical stress from the non-uniform load peak was more directly transferred to the asphalt surface layer, reaching magnitudes close to 1 MPa. This value was substantially higher than the 0.3–0.5 MPa stress typically assumed at this depth under the standard 0.7 MPa uniform contact pressure specified in JTG D50-2017 [17], highlighting the increased vulnerability of underlying structures when thin overlays are used.

### 4.2. Horizontal Transverse Stress Under Varying Thin Layer Thicknesses

**Figure 12:**
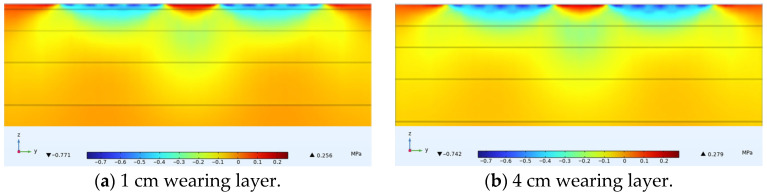
Internal horizontal transverse stress distribution in pavement structure under different thin layer thicknesses.

**Figure 13:**
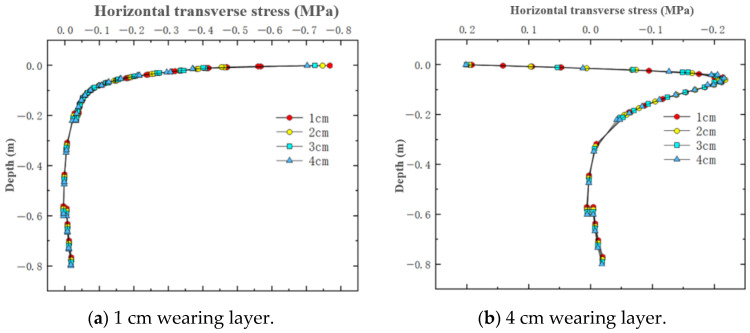
Horizontal transverse stress variation along pavement depth.

**Figure 14:**
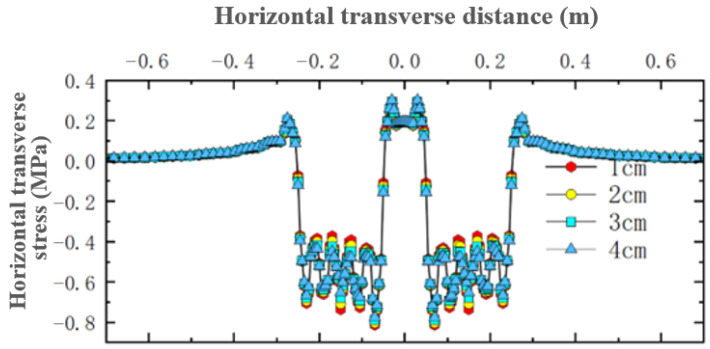
Distribution of surface horizontal transverse stress on cross-section.

For the simulated conditions, horizontal transverse stress is mainly related to longitudinal cracks in the structure. Figure 12 shows the distribution of horizontal transverse stress within thin layer asphalt pavement structures under constant speed loading, showing a cross-section passing through the tire center. Figure 13 shows the distribution of horizontal transverse stress along the pavement depth direction at the load peak point and axle load center. Figure 14 shows the distribution of horizontal transverse tensile stress on a cross-section passing through the tire center.

On the axial line of the tire axle load center, horizontal transverse stress showed a pattern of change from tension to compression in the depth direction. The asphalt layer of ultra-thin wearing course asphalt pavement was mainly characterized by horizontal transverse compressive stress, which gradually decreased with increasing pavement depth. According to simulation results, the maximum horizontal stress within a 4 cm wearing layer is 0.833 MPa, while the maximum horizontal stress within a 1 cm wearing layer is 0.872 MPa. As the thickness increased, this stress peak gradually decreased, with a 3 cm increase reducing it by 4.5%. Within the asphalt surface layer, when the wearing layer thickness was 1 cm, there existed approximately 0.1 MPa of tensile stress and approximately 0.4 MPa of compressive stress, while with a 4 cm thin layer thickness, there was no tensile stress, only approximately 0.2 MPa of compressive stress. Therefore, appropriately increasing the thin layer thickness can reduce the stress on the thin layer and asphalt surface layer, and improve the stress distribution of the entire asphalt pavement structure.

As shown in Figure 14, the pressure of the wheel load caused the areas on both sides of the tire and the inner side areas of the two tires to exhibit tensile states under deformation coordination, with the distribution area of this tensile stress being relatively small. The tensile stress level at the surface was relatively high, approaching 0.3 MPa, and the maximum value was located at the inner edge of the tire load. This surface stress state could easily lead to longitudinal cracks from top to bottom at the inner side of the tire and the axle load center. Comparing four different thin layer thicknesses, it was found that in the areas on both sides of the tire, thin layer thickness had little effect on horizontal tensile stress. However, within the load area, the amplitude of fluctuation in horizontal compressive stress decreased with increasing thickness. It was thus clear that thin layer thickness had a relatively large overall impact on horizontal stress. Within the ultra-thin wearing layer thickness range, appropriately increasing the layer thickness can reduce the amplitude of horizontal transverse stress in the asphalt layer pavement structure.

In summary, the horizontal transverse stress in the ultra-thin wearing layer of asphalt pavement was mainly characterized by horizontal compressive stress, which gradually decreased with increasing pavement depth. The thickness of the thin layer had a relatively large impact on horizontal transverse stress. Reasonably increasing the thin layer thickness can reduce the amplitude of horizontal transverse stress and improve the distribution of horizontal transverse stress.

Crucially, while our analysis identifies the macroscopic stress states that contribute to longitudinal cracking, the actual failure process involves multiscale complexities originating at micro/nanoscales. Crack initiation and propagation are fundamentally controlled by nanomechanical behavior within the asphalt matrix—particularly at aggregate-binder interfacial zones—where localized stress concentrations and material heterogeneity dominate [19]. Mitigating these effects requires advanced material solutions, such as polymer-modified binders engineered for enhanced fatigue resistance and fracture toughness, which demonstrate potential to withstand stress cycles predicted herein [20]. Thus, integrating macroscopic structural analysis with microscale characterization and innovative binder technologies is imperative for developing durable pavement systems.

### 4.3. Horizontal Longitudinal Stress Under Varying Thin Layer Thicknesses

**Figure 15:**
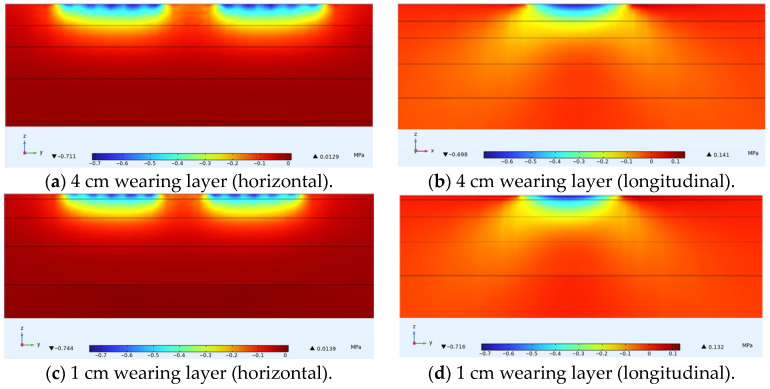
Internal horizontal longitudinal stress distribution in pavement structure under different thin layer thicknesses.

**Figure 16:**
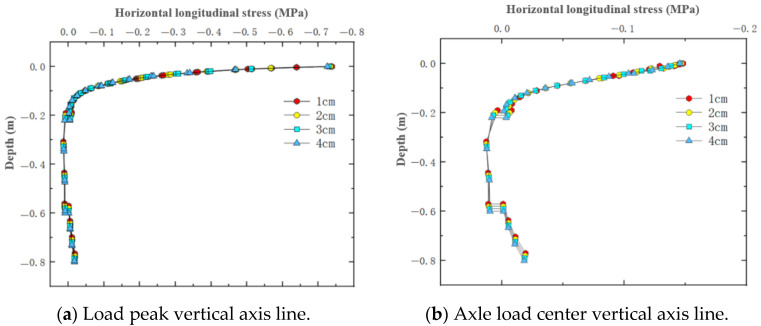
Horizontal longitudinal stress variation along pavement depth.

**Figure 17:**
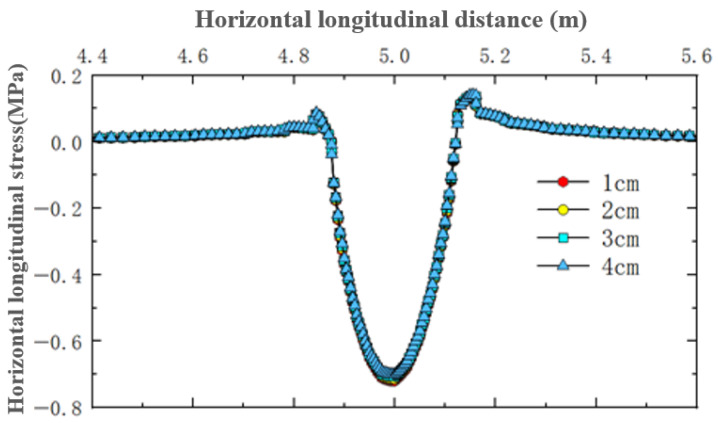
Distribution of surface horizontal longitudinal stress on longitudinal section.

For the simulated conditions, horizontal longitudinal stress is mainly related to transverse cracks in the structure. Figure 15 shows the distribution of horizontal longitudinal stress within thin layer asphalt pavement structures under constant speed loading, showing a horizontal cross-section passing through the tire center and a longitudinal cross-section passing through the load peak point. Figure 16 shows the distribution of horizontal longitudinal stress along the pavement depth direction at the load peak point and axle load center. Figure 17 shows the distribution of horizontal longitudinal tensile stress on a longitudinal section passing through the tire center.

The results showed that on the vertical axis lines of the tire load peak and the tire axle load center, horizontal longitudinal stress exhibited a pattern of change from compression to tension in the depth direction. The asphalt layer of ultra-thin wearing course asphalt pavement was mainly characterized by horizontal longitudinal compressive stress, with relatively small longitudinal tensile stress. As pavement depth increases, longitudinal compressive stress gradually decreased. Comparing the two wearing layer thicknesses in Figure 15, it was found that increasing the wearing layer thickness had little effect on the stress distribution within the wearing layer, but as the thickness decreases, the impact of stress transfer on the asphalt surface layer increases. With a thin layer thickness of 1 cm, the extreme value of longitudinal compressive stress received by the surface layer was approximately 0.5 MPa, while with a thickness of 4 cm, the extreme value of longitudinal compressive stress received by the surface layer was approximately 0.25 MPa. Therefore, when using ultra-thin wearing layers, the asphalt surface layer requires better performance. As shown in Figure 16b, there existed a certain degree of longitudinal tensile stress from the top to the bottom of the structural base layer, with a probability of producing transverse cracks, and the magnitude of this tensile stress had no correlation with the thickness of the wearing layer.

As shown in Figure 17, under constant speed motion, tire rolling gives the pavement a backward friction force, and due to deformation coordination, the tensile stress on the front side was significantly greater among the tensile stresses on both front and rear sides. At the structure surface, wearing layer thickness had no obvious effect on the magnitude of longitudinal horizontal tensile stress on the front and rear sides of the tire load, but it did affect the extreme value of compressive stress in the central load action area. As the wearing layer thickness increased, the compressive stress peak in the load area slightly decreased. It was thus evident that thin layer thickness had a relatively small overall impact on horizontal longitudinal stress, mainly affecting the compressive stress inside the tire load area in the central region of the pavement structure. When setting a 4 cm thin layer, the longitudinal horizontal compressive stress values in the asphalt layer pavement structure are relatively minimal.

In summary, the horizontal longitudinal stress in the ultra-thin wearing layer of asphalt pavement is mainly characterized by longitudinal compressive stress, which gradually decreases with increasing pavement depth. Changes in thin layer thickness have almost no effect on tensile stress, while reasonably increasing thin layer thickness can reduce the amplitude of longitudinal horizontal compressive stress.

### 4.4. Shear Stress Under Varying Thin Layer Thicknesses

**Figure 18:**
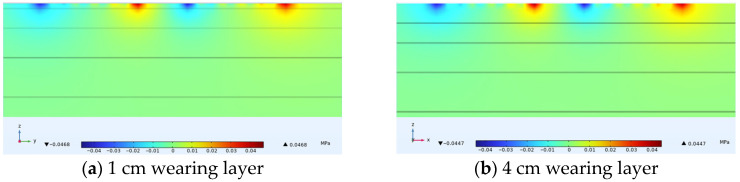
Internal shear stress distribution in pavement structure under different thin layer thicknesses.

**Figure 19:**
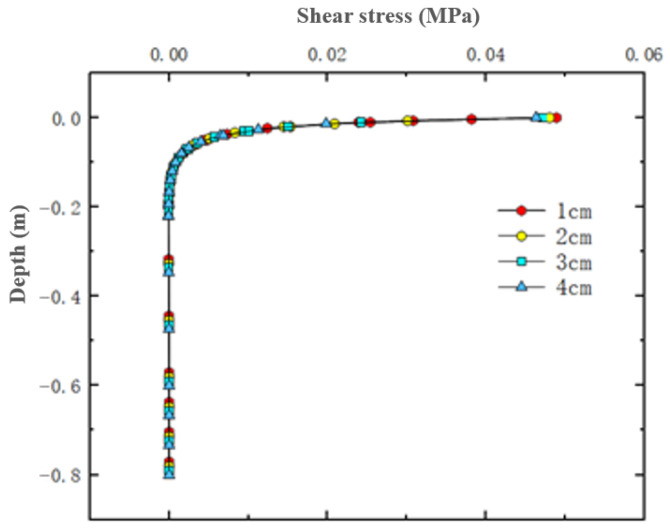
Shear stress variation along pavement depth.

Figure 18 shows the distribution of shear stress on horizontal transverse cross-sections within thin layer asphalt pavement structures under constant speed loading, showing a horizontal cross-section passing through the tire center and a longitudinal cross-section passing through the load peak point. Figure 19 shows the variation of shear stress along the depth direction at the center of the inner edge of the tire load. The results indicated that shear stress attained its maximum value at the pavement surface and progressively diminished with increasing depth. Comparing four different thin layer thicknesses, it was observed that at the wearing layer surface, higher thin layer thicknesses resulted in minimal shear stress. The maximum shear stress for a 4 cm thin layer was 0.0447 MPa and the maximum shear stress for a 1 cm thin layer was 0.0468 MPa. A 3 cm increase in thin layer thickness reduced the maximum shear stress by 4.5%. It was also noted that thinner wearing layers transferred greater shear stress to the asphalt surface layer. With a 4 cm thin layer, the extreme value of shear stress received by the asphalt surface layer was 0.01 MPa, while with a 1 cm thin layer, the extreme value of shear stress received was 0.03 MPa. In summary, reasonably increasing the thickness of the thin layer could improve shear stress distribution and slightly reduce shear stress, but had a relatively small impact on the specific values of shear stress.

## 5. Non-Uniform Load Influence Factor

Based on the aforementioned mechanical analysis results, an empirical model for Non-uniform Load Influence Factor (NLIF) considering thickness effects was established. This model can serve as a reference basis for ultra-thin wearing course structural and material design.

### 5.1. Definition of Influence Factors

Based on the analysis results, it was found that non-uniform loads significantly changed the internal stress state of the ultra-thin wearing layers. To incorporate this influence into structural and material design, this paper proposes a non-uniform load influence factor α, defined as:

(1) $$\alpha =\frac{\sigma_{non-uniform}}{\sigma_{uniform}}$$

where $\sigma_{non-uniform}$ is the extreme value of stress response under non-uniform load conditions, $\sigma_{uniform}$ is the extreme value of stress response under uniform load conditions.

The influence factor reflects the amplification effect of non-uniform loads on the structural stress state. When α > 1, it indicates that non-uniform loads aggravate structural stress; the larger α is, the more significant the influence of non-uniform loads.

### 5.2. Establishment of Empirical Influence Factor Model

Finite element analysis results demonstrated that wearing layer thickness significantly influenced the influence factor α. Through simulation analysis of asphalt pavements with overlaid ultra-thin wearing layers and newly constructed ultra-thin wearing layer asphalt pavements, the extreme values of various stresses under different conditions were obtained, as shown in Table 2. Both uniform and non-uniform loads were studied under eight conditions: asphalt pavements with overlaid ultra-thin wearing layers consisting of 14 cm wearing layer + 4 cm asphalt surface layer + 6 cm asphalt middle layer + 8 cm asphalt bottom layer + 58 cm base layer + 500 cm subgrade, and newly constructed ultra-thin wearing layer asphalt pavements consisting of 14 cm wearing layer + 6 cm asphalt middle layer + 8 cm asphalt bottom layer + base layer + subgrade.

According to the previously defined influence factor, the influence factors for corresponding stresses under each structure were calculated, as shown in Table 3 and Table 4. For example, for the scenario with a 1 cm thick overlay wearing course (i.e., the 1 + 4 + 6 + 8 structure), the calculation procedure for the vertical stress influence factor is as follows:

$$\alpha_{1}=\frac{\sigma_{non-uniform}}{\sigma_{uniform}}=\frac{1.09}{0.79}=1.3797468354$$

For the scenario of a newly constructed road with a 1 cm thick wearing course (i.e., the 1 + 6 + 8 structure), the calculation procedure for its vertical stress influence factor is as follows:

$$\alpha_{2}=\frac{\sigma_{non-uniform}}{\sigma_{uniform}}=\frac{1.09}{0.789}=1.3814955640$$

Variation of influence factors for various stresses were calculated, as shown in Table 5.

**Figure 20:**
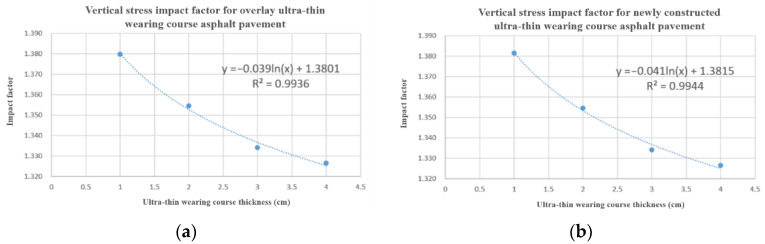

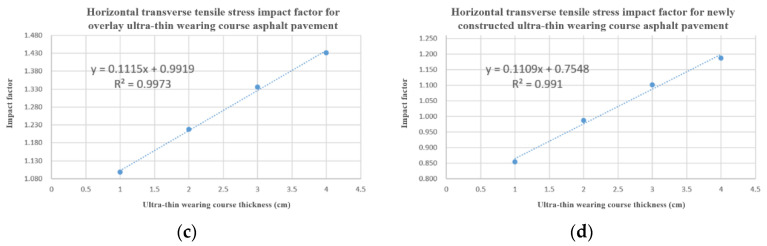
Regression analysis of influence factor vertical stress and horizontal transverse tensile stress. (**a**) Vertical stress influence factor for overlay condition. (**b**) Vertical stress influence factor for new construction condition. (**c**) Horizontal transverse tensile stress influence factor for overlay condition. (**d**) Horizontal transverse tensile stress influence factor for new construction condition.

Observing the above influence factor values, it was found that the variations (max-min difference) of influence factors for horizontal transverse compressive stress, horizontal longitudinal tensile and compressive stress, and shear stress were all below 0.04, with the variation of longitudinal tensile stress and shear stress being less than 0.01. Therefore, the influence factors for these four stresses were considered stable enough for direct use. In contrast, regression analysis was performed for vertical stress and horizontal transverse tensile stress, where the variation was more significant, with the results depicted in Figure 20.

Through regression analysis of vertical stress for both conditions, a vertical stress influence factor formula applicable to both conditions was obtained:

(2) $$\alpha_{V}=-0.04\ln(h)+1.38\;(R^{2}=0.99)$$

where $\alpha_{V}$ represents the vertical stress influence factor, h is thickness of ultra-thin wearing layer (cm).

For horizontal transverse tensile stress, as shown in Table 3 and Table 4, under the same ultra-thin wearing layer thickness, the horizontal transverse tensile stress influence factors for overlay conditions were all greater than those for new construction conditions. Therefore, unlike the vertical stress condition, the regression formulas for the two conditions could not be unified. Horizontal transverse stress influence factor formula:

(3) $$\alpha_{HTt}=\left\{\begin{array}{c} 0.1115h_{1}+0.9919\\ 0.1109h_{2}+0.7548 \end{array}\right.(R^{2}=0.99)$$

where $\alpha_{HTt}$ represents $\alpha_{Horizontal\_Transverse\_tensile}$, the horizontal transverse tensile stress influence factor. h_1_ is the overlay condition ultra-thin wearing layer thickness (cm), h_2_ is the new construction condition ultra-thin wearing layer thickness (cm).

From a conservative pavement structure design perspective, the horizontal transverse stress influence factor formulas for both conditions were combined by taking the larger value and simplified, resulting in a combined horizontal transverse stress influence factor formula:

(4) $$\alpha_{HTt}=0.11h+1$$

where $\alpha_{HTt}$ represents the horizontal transverse tensile stress influence factor, h is the ultra-thin wearing layer thickness (cm).

For horizontal transverse compressive stress, horizontal longitudinal tensile and compressive stress, and shear stress, since the extreme differences were small and the influence factors were only minimally affected by changes in wearing layer thickness, direct values were taken from the data in Table 3 and Table 4. Finally, non-uniform load influence factors based on ultra-thin wearing layer thickness were obtained from Equations (2) and (4) and Table 3 and Table 4, as shown in Table 6.

Where: h is the ultra-thin wearing layer thickness (cm). $\alpha_{V}$ represents $\alpha_{Vertical}$, the vertical stress impact factor. $\alpha_{HTc}$ represents $\alpha_{Horizontal\_Transverse\_compressive}$, the horizontal transverse compressive stress impact factor. $\alpha_{HTt}$ represents $\alpha_{Horizontal\_Transverse\_tensile}$, the horizontal transverse tensile stress impact factor. $\alpha_{HLc}$ represents $\alpha_{Horizontal\_Longitudinal\_compressive}$, the horizontal longitudinal compressive stress impact factor. $\alpha_{HLt}$ represents $\alpha_{Horizontal\_Longitudinal\_tensile}$, the horizontal longitudinal tensile stress impact factor. $\alpha_{\tau }$ represents the shear stress impact factor.

### 5.3. Design Recommendations

Based on the aforementioned non-uniform load influence factor analysis results, combined with the relevant requirements of Standard Test Methods of Bitumen and Bituminous Mixtures for Highway Engineering (JTG E20-2011) [21] and Specifications for Design of Highway Asphalt Pavement (JTG D50-2017) [17], modification suggestions for ultra-thin wearing course asphalt mixture design and testing are proposed. Current specifications simulate the actual tire contact surface through rectangular uniform loads, using a 0.7 MPa standard wheel pressure for design and evaluation. The findings of this study revealed that non-uniform loads significantly amplified the stress state within ultra-thin wearing layers, with vertical stress peaks increasing by over 35%. This finding has direct implications for current design practices. Since traditional methods based on uniform load assumptions may underestimate the actual stress levels, they could lead to an unconservative design and premature pavement failure. Therefore, it is necessary to make appropriate modifications to the material evaluation methods for ultra-thin wearing layers.

Specifications for Design of Highway Asphalt Pavement uses cumulative permanent deformation to control rutting development. Based on rutting tests under standard conditions, the permanent deformation of each sublayer is calculated:

(5) $$R_{a}=\sum_{i=1}^{n}R_{ai}$$

(6) $$R_{ai}=2.31\times 10^{-8}k_{Ri}T_{pef}^{2.93}p_{i}^{1.80}N_{e3}^{0.48}(h_{i}/h_{0})R_{0i}$$

$R_{0i}$ is the permanent deformation from rutting tests of the asphalt mixture in the i-th sublayer (mm), $p_{i}$ is the vertical compressive stress at the top of the i-th sublayer of the asphalt mixture layer. The permanent deformation calculation formula includes a load term $p_{i}^{1.80}$, which relationship is obtained through regression analysis of a large amount of experimental data, indicating that there is a nonlinear relationship between compressive stress and permanent deformation in asphalt pavement structures. The non-uniform load influence factor defined in this study represents the ratio of stress response under non-uniform load to stress response under uniform load. The deformation of asphalt mixtures is directly related to the stress state and follows the aforementioned nonlinear relationship. Therefore, it can be deduced that the permanent deformation increment under non-uniform loads is positively correlated with $\alpha_{V}^{1.80}$.

According to Standard Test methods of Bitumen and Bituminous Mixtures for Highway Engineering, the dynamic stability of asphalt mixtures is calculated using the following formula:

(7) $$DS=\frac{(t_{2}-t_{1})\times N}{d_{2}-d_{1}}\times C_{1}\times C_{2}$$

where, DS is the dynamic stability of the asphalt mixture (cycles/mm), $d_{1}$ is the deformation at time $t_{1}$ (mm), $d_{2}$ is the deformation at time $t_{2}$ (mm), N is the reciprocating rolling speed of the test wheel, $C_{1}$ is the test machine type coefficient, $C_{2}$ is the specimen coefficient.

Considering the influence of non-uniform loads, the dynamic stability calculation formula is modified to:

(8) $$DS=\frac{(t_{2}-t_{1})\times N}{\alpha \times (d_{2}-d_{1})}\times C_{1}\times C_{2}$$

α is the non-uniform load influence factor, calculated by:

(9) $$\alpha =C_{3}\times \alpha_{V}^{1.80}$$

where, $C_{3}$ is the graded correction factor, valued based on highway and road section classification, $\alpha_{V}$ is the non-uniform load influence factor corresponding to vertical stress, determined according to the formula in Table 6. 1.80 is the exponent considering the nonlinear relationship between load and deformation, reflecting the nonlinear effect of accelerated material deformation under load, which is consistent with the deformation mechanism of asphalt mixtures and maintains consistency with the permanent deformation calculation model in the specifications.

For formula (9), when the graded correction factor $C_{3}$ is 1 and a 1 cm ultra-thin wearing layer is used, according to Table 6, $\alpha_{V}=1.38$, $\alpha_{V}^{1.80}=1.38^{1.80}=1.78559$, which means that the modified dynamic stability is about 56% of the original calculated value. Although this modification amplitude was relatively large, it was basically consistent with the theoretical value after nonlinear amplification of the stress increase (35.4%) caused by non-uniform loads. As the wearing layer thickness increases, the influence of non-uniform loads gradually decreases. For example, for a 4 cm thick wearing layer, $\alpha_{V}=-0.04\times \ln(4)+1.38\approx 1.33$, $\alpha_{V}^{1.80}=1.33^{1.80}=1.67083$. The modified dynamic stability is about 59% of the original value, with a smaller decrease than in the 1 cm thickness case. This thickness-related trend was consistent with the finite element analysis results, which indicated that the modification method can reasonably reflect the mechanical response characteristics of ultra-thin wearing layers of different thicknesses.

Considering that the modification amplitude may be relatively large, to ensure the rationality of engineering applications, a correction factor $C_{3}$ is introduced. For high-grade highways, heavy-load traffic sections, and areas with severe climate conditions, it is recommended to adopt the complete correction coefficient, i.e., $C_{3}$ = 1, to ensure the safety margin of the design. For medium and low-grade highways or general road sections with lighter traffic loads, the correction amplitude can be appropriately adjusted according to specific circumstances, for example, with a graded correction factor $C_{3}$ of 0.8, balancing safety and economy.

More broadly, the application of the NLIF provides a quantitative tool for engineers to move beyond simplified assumptions and assess the true stress state within the pavement. This realistic assessment is crucial for material selection and specification. For instance, a design scenario yielding a higher stress level after applying the NLIF may justify the selection of higher-performance materials, such as polymer-modified binders or stone matrix asphalt (SMA) mixtures, which are known for their superior fatigue and rutting resistance. Ultimately, this approach bridges the gap between mechanistic analysis and practical material engineering, contributing to more durable and reliable pavement designs that aim to maximize longevity and minimize life-cycle costs.

## 6. Conclusions

This study systematically investigated the mechanical response characteristics of ultra-thin asphalt wearing courses under non-uniform axial loading conditions, yielding the following main conclusions:

(1) Compared with traditional uniform load assumptions, non-uniform loading pressure significantly altered the internal stress distribution within the ultra-thin wearing layer. This study found that vertical stress peaks increased by 35.4% and horizontal transverse compressive stress increased by 22.1%. Moreover, this stress amplification became more prominent as the wearing course thickness decreased. These results indicated that existing load simplification methods significantly underestimated the actual stress levels in ultra-thin wearing courses.
(2) The model simulation results for varied wearing layer thicknesses showed that as the thickness decreased, the influence of non-uniform loading pressure became more significant. When the thickness reached 1 cm, local stress concentration was particularly pronounced. This finding provided important insight for the thickness optimization design of ultra-thin wearing courses.
(3) Based on numerous numerical simulation results, a Non-uniform Load Influence Factor (NLIF) that considers thickness effects was established, along with corresponding design recommendations. This model enables practical engineering applications by modifying the dynamic stability calculation formula for asphalt mixture rutting tests, effectively transforming non-uniform load effects from the pavement structure scale to the material test scale. As such, it allows for the consideration of non-uniform loading influences through calculation adjustments without requiring changes to existing test equipment and procedures.

Future investigations should prioritize:

(1) Field validation via micro-strain sensors embedded in 1–2 cm ultra-thin layers.
(2) Multiphysics integration of thermo-mechanical effects and moisture-induced damage.
(3) Standardization pathways for NLIF adoption in AASHTO/JTG design frameworks.

The research findings provided theoretical guidance for the structural design and material optimization of ultra-thin wearing course pavements, thereby contributing to the potential for improved design standards and an extended service life for these pavement structures.

## Figures and Tables

**Table 1:** Thickness and material parameter values for pavement structural layers.

Structural Layer	Thickness (cm)	Modulus (MPa)	Poisson’s Ratio
Ultra-thin Layer	1, 2, 3, 4	11,000	0.25
Asphalt Surface Layer	4	10,375	0.25
Asphalt Middle Layer	6	11,224	0.25
Asphalt Bottom Layer	8	11,197	0.25
Upper Base	38	13,000	0.25
Lower Base	20	9000	0.25
Subgrade	500	40	0.4

**Table 2:** Extreme values of various stresses under different conditions.

Load	Condition	Surface Layer Structure (cm)	Vertical Stress (MPa)	Horizontal Stress (MPa)				Shear Stress (MPa)
				Transverse Compressive Stress	Transverse Tensile Stress	Longitudinal Compressive Stress	Longitudinal Tensile Stress	
Uniform Load	Overlay	1 + 4 + 6 + 8	0.79	0.71	0.142	0.797	0.151	0.0451
		2 + 4 + 6 + 8	0.79	0.705	0.138	0.794	0.147	0.0445
		3 + 4 + 6 + 8	0.787	0.699	0.134	0.788	0.143	0.0439
		4 + 4 + 6 + 8	0.784	0.694	0.13	0.782	0.139	0.0433
	New Construction	1 + 6 + 8	0.789	0.708	0.158	0.795	0.168	0.0438
		2 + 6 + 8	0.79	0.707	0.153	0.794	0.163	0.0437
		3 + 6 + 8	0.787	0.704	0.149	0.792	0.158	0.0435
		4 + 6 + 8	0.784	0.699	0.145	0.788	0.154	0.043
Non-uniform Load	Overlay	1 + 4 + 6 + 8	1.09	0.872	0.156	0.821	0.123	0.0468
		2 + 4 + 6 + 8	1.07	0.861	0.168	0.813	0.12	0.0461
		3 + 4 + 6 + 8	1.05	0.847	0.179	0.804	0.116	0.0454
		4 + 4 + 6 + 8	1.04	0.833	0.186	0.795	0.113	0.0447
	New Construction	1 + 6 + 8	1.09	0.863	0.135	0.823	0.137	0.0456
		2 + 6 + 8	1.07	0.856	0.151	0.816	0.133	0.0454
		3 + 6 + 8	1.05	0.848	0.164	0.807	0.129	0.0451
		4 + 6 + 8	1.04	0.837	0.172	0.799	0.126	0.0445

**Table 3:** Influence factors for various stresses of overlay ultra-thin wearing layer asphalt pavement with different wearing layer thicknesses.

Wearing Layer Thickness (cm)	Vertical Stress	Horizontal Stress				Shear Stress
		Transverse Compressive Stress	Transverse Tensile Stress	Longitudinal Compressive Stress	Longitudinal Tensile Stress	
1	1.3797468354	1.2281690141	1.0985915493	1.0301129235	0.8145695364	1.0376940133
2	1.3544303797	1.2212765957	1.2173913043	1.0239294710	0.8163265306	1.0359550562
3	1.3341804320	1.2117310443	1.3358208955	1.0203045685	0.8111888112	1.0341685649
4	1.3265306122	1.2002881844	1.4307692308	1.0166240409	0.8129496403	1.0323325635

**Table 4:** Influence factors for various stresses of new construction ultra-thin wearing layer asphalt pavement with different wearing layer thicknesses.

Wearing Layer Thickness (cm)	Vertical Stress	Horizontal Stress				Shear Stress
		Transverse Compressive Stress	Transverse Tensile Stress	Longitudinal Compressive Stress	Longitudinal Tensile Stress	
1	1.3814955640	1.2189265537	0.8544303797	1.0352201258	0.8154761905	1.0410958904
2	1.3544303797	1.2107496464	0.9869281046	1.0277078086	0.8159509202	1.0389016018
3	1.3341804320	1.2045454545	1.1006711409	1.0189393939	0.8164556962	1.0367816092
4	1.3265306122	1.1974248927	1.1862068966	1.0139593909	0.8181818182	1.0348837209

**Table 5:** Variation (max-min difference) of influence factors for various stresses of ultra-thin wearing layer asphalt pavement.

Vertical Stress	Horizontal Stress				Shear Stress
	Transverse Compressive Stress	Transverse Tensile Stress	Longitudinal Compressive Stress	Longitudinal Tensile Stress	
0.0549649518	0.0307441214	0.5763388510	0.0212607349	0.0069930070	0.0087633269

**Table 6:** Non-uniform load influence factors based on ultra-thin wearing layer.

Corresponding Stress	Corresponding Formula
Vertical Stress	$\alpha_{V}=-0.04\ln(h)+1.38$
Horizontal Transverse Compressive Stress	$\alpha_{HTc}=1.23$
Horizontal Transverse Tensile Stress	$\alpha_{HTt}=0.11h+1$
Horizontal Longitudinal Compressive Stress	$\alpha_{HLc}=1.03$
Horizontal Longitudinal Tensile Stress	$\alpha_{HLt}=0.82$
Shear Stress	$\alpha_{\tau }=1.04$

## Data Availability

The original contributions presented in this study are included in the article. Further inquiries can be directed to the corresponding author.
